# Pre-Clinical Pharmacokinetic Characterization, Tissue Distribution, and Excretion Studies of Novel Edaravone Oral Prodrug, TEJ-1704

**DOI:** 10.3390/pharmaceutics13091406

**Published:** 2021-09-04

**Authors:** Dong Wook Kang, Ju Hee Kim, Kyung Min Kim, Seok-jin Cho, Hee-Woon Jang, Ji Won Chang, Seung Myung Dong, Jee Woong Lim, Jae-Sun Kim, Hea-Young Cho

**Affiliations:** 1 College of Pharmacy, CHA University, 335 Pangyo-ro, Seongnam 13488, Korea; dongwk203@gmail.com (D.W.K.); 20135107@ppharm.org (J.H.K.); mini805mini@gmail.com (K.M.K.); sj20219106@gmail.com (S.-j.C.); heewoonnang@naver.com (H.-W.J.); 2 Theragen Etex Co., Ltd., 190 Gangnam-daero, Seoul 06744, Korea; jiwon.chang@therabio.kr (J.W.C.); smdong@etexpharm.com (S.M.D.); 3 J2HBiotech Inc., 142-10 Saenam-ro, Suwon 16648, Korea; jw0120@j2hbio.com (J.W.L.); jsbach@j2hbio.com (J.-S.K.)

**Keywords:** pharmacokinetics, tissue distribution, excretion, edaravone oral prodrug, amyotrophic lateral sclerosis

## Abstract

Edaravone (3-methyl-1-phenyl-2-pyrazolin-5-one) is a free radical scavenger approved for the treatment of amyotrophic lateral sclerosis, a fatal neuromuscular disease. Edaravone is administered as an intravenous infusion over 60 min for several treatment cycles. To ease the burden of patients and caregivers, the oral formulation of edaravone has been developed. The purpose of this study was to evaluate pharmacokinetics and tissue distribution of TEJ-1704, an edaravone oral prodrug, in male Sprague Dawley rats and beagle dogs. Animal experiments were conducted using Sprague Dawley rats and beagle dogs to evaluate pharmacokinetics, tissue distribution, and excretion of TEJ-1704. Blood, tissues, cerebrospinal fluid, urine, and feces samples were collected at designated sampling time after intravenous (IV) or oral (PO) administration of edaravone or TEJ-1704. A modified bioanalysis method was developed to quantify edaravone in samples including plasma, tissues, cerebrospinal fluid, urine, and feces. The bioanalysis method was validated and successfully applied to pharmacokinetics, tissue distribution, and excretion studies of the novel edaravone prodrug. Although plasma C_max_ of TEJ-1704 was low, groups administered with TEJ-1704 had high AUC_inf_, suggesting continuous metabolism of TEJ-1704 into edaravone. Groups treated with TEJ-1704 also showed lower CSF distribution than the control groups. After the administration of TEJ-1704, the majority of edaravone was distributed to the heart, lung, and kidney. It was excreted equally via urine and feces. The pharmacokinetics, tissue distribution, and excretion of TEJ-1704, a novel edaravone oral prodrug, were successfully characterized. Additional studies are needed to fully understand the difference between TEJ-1704 and edaravone and determine the potency of TEJ-1704.

## 1. Introduction

Amyotrophic lateral sclerosis (ALS) is a disorder that causes degeneration of cerebral and spinal cord motor neurons. Although its disease progression rate greatly differs depending on individuals, death occurs within 3–5 years after its diagnosis without ventilator support [1]. The underlying cause of ALS remains unclear. Several studies have reported that the concentration of 3-nitrotyrosin (3-NT, a marker of oxidative stress in the spinal cord) and 3-NT immunoreactivity in motor neurons of sporadic and familial ALS patients are increased, suggesting that oxidative stress is an essential factor associated with ALS [2].

Edaravone (3-methyl-1-phenyl-2-pyrazolin-5-one), a novel antioxidant drug, is approved for treating ALS in several countries including South Korea, United States, and Japan. Although its mechanism of action remains unclear, it has been reported that edaravone can prevent motor neuron death caused by oxidative stress, inhibit tyrosine residue nitration in cerebrospinal fluid (CSF), and improve motor functions in vivo [3,4]. Edaravone is sold under the brand name Radicava or Radicut. The recommended clinical dose is 60 mg of edaravone administered via IV infusion over 60 min. According to the US FDA drug label, the initial treatment cycle requires daily dosing of edaravone 60 mg for 14 days followed by a 14-day drug-free period with subsequent treatment cycles of daily dosing of edaravone 60 mg for 10 days out of a 14-day period, followed by a 14-day drug-free period. As its approved route of administration and treatment cycle greatly burden ALS patients and caregivers, an edaravone oral formulation is needed [1].

The development of TEJ-1704, an edaravone oral prodrug, was devised to improve the discomfort of invasive administration and extend the duration of action. Moreover, we conducted a pharmacokinetic evaluation and tissue distribution study of TEJ-1704 in rats and beagle dogs. To evaluate a PK of edaravone after administration of TEJ-1704, a sensitive quantitation method for edaravone in rat and beagle dog matrices is needed. The bioanalysis method in HPLC and LC-MS/MS have been reported in rat plasma [5,6], organs including kidney, heart, brain, spleen, liver [7], bile, urine, and feces [8]. Aside from Li et al. [7], all the other methods had lower sensitivity with the lower limit of quantification (LLOQ) of 100 ng/mL but required rather large volume of matrices (100 μL). Although Li et al. have lowered LLOQ drastically to 2 ng/mL, it was only validated in 5 organ matrices. Yu [9] and Shao [10] have each developed and validated quantitation methods for edaravone in beagle plasma with LLOQ of 50 and 10 ng/mL, respectively. However, there was no report on the bioanalysis of edaravone in beagle CSF, urine, or feces.

In this study, a modified bioanalysis method using LC-MS/MS was developed and validated for pharmacokinetic evaluation of edaravone after IV and PO administration of edaravone or TEJ-1704 in rats and beagle dogs. Furthermore, in vitro and in vivo efficacy of TEJ-1704 were evaluated. To further observe the distribution and excretion of edaravone after TEJ-1704 administration, six major organs (brain, heart, lung, kidney, liver and gastrointestinal tract) in rats and CSF, urine, and feces in beagle dogs were collected for the analysis.

## 2. Materials and Methods

### 2.1. Chemicals and Reagents

**Figure 1:**
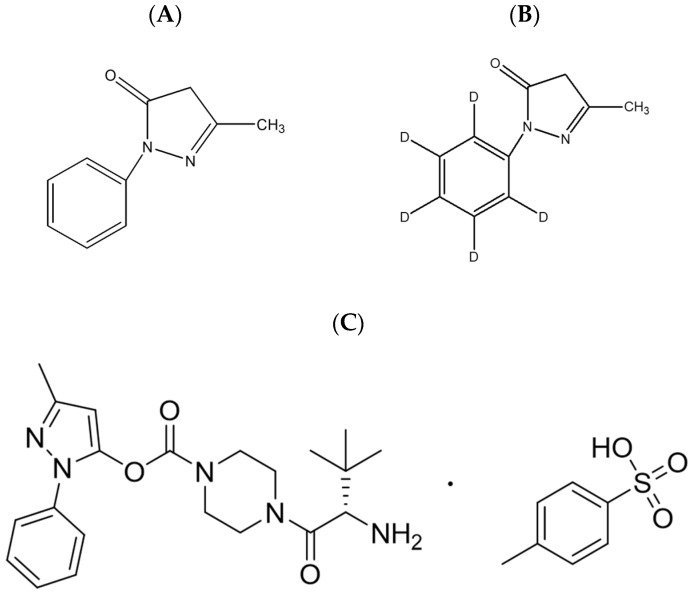
Chemical structures of (**A**) edaravone and (**B**) edaravone-d5 (IS) (**C**) TEJ-1704.

TEJ-1704, 3-methyl-1-phenyl-1H-pyrazol-5-yl (S)-4-(2-amino-3,3-dimethylbutanoyl)piperazine-1-carboxylate p-toluensulfonic acid, was prepared by J2H Biotech Inc. (Suwon, Korea). Reference standard for edaravone (CAS No. 89-25-8) and edaravone-d5 (CAS No. 1228765-67-0; Internal standard, IS) was purchased from Toronto Research Chemicals Inc. (Toronto, ON, Canada), and their chemical structures are presented in Figure 1. LC-MS grade acetonitrile, methyl tert-butyl ether (MTBE) and methanol were purchased from J.T. Baker Inc. (Phillipsburg, NJ, USA). Sodium metabisulfite (SMB), trichloroacetic acid (TCA), formic acid, DPPH (1,1-diphenyl-2-picrylhydrazyl radical), and quercetin dehydrate were purchased from Sigma-Aldrich (St. Louis, MO, USA). All chemicals had the highest HPLC grade or quality available. Heparin sodium (25000 IU/5 mL) was purchased from Choongwae Pharm. Corp. (Seoul, Korea). Fasted and pooled male Sprague Dawley rat plasma in sodium heparin was purchased from BioChemed (Winchester, VA, USA).

### 2.2. Preparation of TEJ-1704

TEJ-1704 has a molecular weight of 571.70 g/mol and a molecular formula of C_28_H_37_N_5_O_6_S. Synthesis of TEJ-1704 consists of five steps as follows. In the first step chloroformylation of N-piperazine takes place between N-Boc-piperazine and triphogene yielding the first intermediate tert-butyl 4-(chlorocarbonyl)piperazine-1-carboxylate. In the second step conjugation between the first intermediate and edaravone offers the second intermediate 1-(tert-butyl) 4-(3-methyl-1-phenyl-1H-pyrazol-5-yl) piperazine-1,4-dicarboxylate using cesium carbonate. The next step, deprotection of N-BOC of piperazine takes place with HCl in isopropanol solution yielding the intermediate 3-methyl-1-phenyl-1H-pyrazol-5-yl piperazine-1-carboxylate as a HCl salt. The following amide coupling reaction with (S)-2-((tert-butoxycarbonyl)amino)-3,3-dimethylbutanoic acid, which is a key step in overall process, is performed with diisopropylcarbodiimide as a coupling agent in aprotic polar solvent DMF. In the last step deprotection of N-BOC of t-leucine moiety is performed in acidic condition followed by crystallization from isopropanol for the purification to offer the final product TEJ-1704 as an HCl salt. The structure of TEJ-1704 was identified by proton NMR and mass spectrometry. 1H NMR (400 MHz, DMSO-d6) δ 0.95 (9H, s), 2.18 (3H, s), 3.30–3.60 (8H, m), 4.20 (1H, s), 6.08 (1H, s), 7.31–7.51 (5H, m), 8.14 (3H, br s). [M + 1]+, 400.1. TEJ-1704 HCl salt shows good solubility in water but is strongly hygroscopic because of lack of crystallinity. Therefore, we performed salt screening study of TEJ-1704 using several organic acids, for example, benzenesulfonic acid, p-toluenesulfonic acid, and methanesulfonic acid to obtain desirable physicochemical properties. After repeated preparation of acid salts of TEJ-1704, we found very stable solid form as a tosylate (p-toluenesulfonate) salt not showing hygroscopicity. The synthetic process of TEJ-1704 tosylate is as follows. TEJ-1704 HCl salt was neutralized with sodium bicarbonate aq. solution and the resultant free TEJ-1704 was extracted by ethyl acetate. After drying with anhydrous magnesium sulfate, a solution of p-toluenesulfonic acid in ethyl acetate (1 equivalent) was added portion wise to make white solid suspension. Filtration and further drying in vacuo offered TEJ-1704 tosylate as a good crystalline.

### 2.3. In Vitro Assessment of Antioxidant Activity

Total of 0.2 mM DPPH solution was put into a 96-well plate at 190 μL per well, and 10 μL of the test substance (edaravone or TEJ-1704) was added to each well so that the final concentrations were 6.25, 12.5, 25, 50, and 100 μM. The control group was injected with 10 μL of DMSO instead of the test substance, and the positive control group was injected with 10 μL of quercetin (so that the final concentration was 20 μm). The reaction was carried out at room temperature for 1 h, and absorbance was then measured at 517 nm using a microplate reader (SUNRISE Tecan, Grödig, Austria).

### 2.4. In Vivo Efficacy Study

The in vivo efficacy of edaravone and TEJ-1704 was evaluated using the ALS animal model, SOD1 G93A transgenic mouse (12 weeks, 24 males and 24 females). This study was approved by MEDIFRON DBT Inc., Republic of Korea (approval number: Medifron 2019-13). Rota-Rod (Ugo Basile, Comerio, italy) was used to determine the onset timing of motor function deficit in SOD1 mice. The rotation speed was set at 15 rpm and the experiment was carried out for up to 300 s. The rotarod test was conducted twice a week, with *n* = 3 was performed for each test. The onset of motor function deficit was defined as failure to maintain 300 s for two consecutive times in three trials of each test. After the onset of motor function deficit, the test substance was administered, and survival rates were measured for 143 or 150 days. Distilled water (vehicle), edaravone (15 mg/kg), and TEJ-1704 (75 and 150 mg/kg) were each administered to SOD 1 mice (*n* = 5 each). Each compound was administered daily from the onset of motor function deficit, and the death of the mice was checked every day.

### 2.5. Animals and Pharmacokinetic Study Design

The Sprague Dawley (SD) rat study was approved by CHA University Animal Experimental Ethics Committee, Republic of Korea (approval number: IACUC200007). Male SD rats (7 weeks, 222–271 g) were purchased from Orient Bio Inc. (Seongnam, Republic of Korea). All rats were separately housed in cages in a ventilated animal room with a temperature of 23 ± 1 °C, a relative humidity of 50 ± 5%, and a 12-h/12-h of light/dark cycle. Rats were freely accessible to food and water, but they were fasted overnight before the drug administration. For pharmacokinetic studies, rats were randomly allocated into nine groups. The pharmacokinetic study design is summarized in Table 1. In R1-R9 groups, blood samples (200 μL) were drawn from the jugular vein into tubes treated with heparin and 10% TCA. The 10% TCA was used as an antioxidant stabilizer that improves the plasma stability of edaravone [10]. Blood samples were centrifuged at 13,000 rpm for 10 min followed by being transferred into new tubes and stored at −80 °C until the analysis. Except for the tosylate salt and other moieties from the prodrug, the edaravone equivalent doses were calculated and are summarized in Table 1 and Table 2.

Rats were randomly allocated into three groups (18 rats/group) for tissue distribution studies (R7, R8, and R9). These rats were sacrificed at 15, 30, 45 min and 1, 2, 4 h after orally administering TEJ-1704. Six major organs including brain, heart, lung, kidney, liver, and gastrointestinal tract were immediately removed, washed in normal saline, and dried with filter papers. Organs were accurately weighed and individually homogenized with distilled water (organ: distilled water = 1:4 *w*/*v*). The tissue homogenate was stored at −80 °C until analysis.

The beagle dog study was approved by Knotus Co., Ltd. Animal Experiment Ethics Committee (approval number: 20-KE-318), Korea. All dogs were separately housed in cages in a ventilated animal room with a temperature of 23 ± 3 °C, a relative humidity of 55 ± 15%, and a 12-h/12-h of light/dark cycle. Dogs were freely accessible to food and water, but they were fasted overnight before the drug administration. Male beagle dogs (13 months, average 9.4 kg) were randomly divided into six groups (5 dogs/group) for the pharmacokinetic study (Table 2). In group B1, the administration route of edaravone was set as an IV infusion in the same way as the clinical route. Blood and CSF samples were collected into tubes treated with heparin and 10% TCA. Urine and fecal samples were collected using metabolic cages for the excretion study. After oral administration of edaravone or TEJ-1704, urine and feces were collected at 0–2, 2–6, 6–12, 12–24 h. Urine volumes were recorded and transferred to centrifuge tubes (1 mL each) for use as urine samples. Fecal weights were recorded after thoroughly mixing feces collected at each sampling period. Then 1 g of feces was accurately weighed, mixed with 4 mL of distilled water, and homogenized to be used as a fecal sample. All samples were stored at −80 °C until analysis.

### 2.6. Analytical Methodology

A modified bioanalysis method was developed for edaravone based on several references [6,9,10] using an Agilent 1290 Infinity II UHPLC system coupled with Agilent 6490 triple quadrupole mass spectrometer (Agilent Technologies Inc., Santa Clara, CA, USA). Edaravone and IS were accurately weighed and dissolved in methanol at a concentration of 1 mg/mL prior to making working solutions. The stock solution was stored at −20 °C. Standard working solutions for edaravone (0.02, 0.05, 0.5, 2, 10, and 20 μg/mL) and IS (20 ng/mL) were diluted with 50% methanol from their standard stock solutions. Then 5 μL of 20% formic acid was added to each working solution to increase the stability of edaravone. To obtain calibration standards, the working solution was mixed with blank rat plasma, urine, and feces so that final concentrations of edaravone ranged from 2 to 2000 ng/mL. Individual calibration curves were prepared for plasma, urine, and feces. For accuracy and precision, quality control (QC) samples of edaravone at four final concentrations (2, 6, 800, 1600 ng/mL) were similarly prepared. The preparation of calibration and QC samples were conducted on the day of analysis.

The sample preparation for determining edaravone was performed using protein precipitation [11] method and liquid–liquid extraction (LLE) method. Total of 150 μL of acetonitrile was added to 50 μL of each sample and 10 μL of working IS solution (20 ng/mL edaravone-d5 diluted with 50% methanol). The mixture was vortexed (VortexGenie 2 Mixer, Scientific Industries Inc., Bohemia, NY, USA) for 3 min and then centrifuged (Centrikon T-2070, Kontron, Switzerland) at 15,000× *g* for 10 min at 4 °C. Then 80 μL of the supernatant organic layer was collected and mixed with 160 μL of methyl tert-butyl ether and 10 μL of 10% TCA, which was then vortexed for 3 min and centrifuged at 15,000× *g* for 10 min at 4 °C. Finally, 200 μL of the supernatant was dried with a nitrogen evaporator (CVE-3000, EYELA Corp., Tokyo, Japan) at room temperature. The dried matter was reconstituted with 75 μL of acetonitrile and water (45/55, *v*/*v*) and vortexed for 3 min. After centrifugation at 15,000× *g* for 5 min at 4 °C, 5 μL of the aliquot was injected for UHPLC MS/MS analysis.

Optimal chromatographic separation of edaravone was conducted using an Acclaim Polar Ad-vantage II C18 column (2.1 × 100 mm, 3 μm, Agilent Technologies Inc., Santa Clara, CA, USA) with a binary isocratic mobile phase. The mobile phase was prepared with distilled water (mobile phase A) and acetonitrile (mobile phase B) (A:B = 55:45). The flow rate was 0.2 mL/min. The autosampler tray temperature and the column temperature were maintained at 4 °C and 40 °C, respectively. The total run time per sample was 5 min. The analysis was conducted with positive electrospray ionization (ESI). Quantification was achieved using MRM mode at m/z 175.10 → 133.00 for edaravone and at m/z 180.12 → 80.90 for IS. Acquisition and analysis of data were performed using a Masshunter Workstation version B.07 (Agilent Technologies Inc., Santa Clara, CA, USA) at collision energy 35 for edaravone and 45 for IS. Capillary voltage of 3000 V, gas temperature of 200 °C, drying nitrogen gas flow at 14 L/min, and nebulizer of 20 psi were used. The dwell time was 100 msec for all compounds. The developed method was validated in accordance with the FDA guidance [12].

### 2.7. Pharmacokinetic Evaluation

A non-compartmental analysis was performed using a WinNonLin software (version 8.2, Certara^TM^ Company, NJ, USA). The elimination rate constant (k) was estimated from the slope (−k/2.303) of the terminal log-linear phase of the plasma concentration–time curve and elimination half-life (t_1/2_) was calculated as following Equation (1):

(1) $$t_{1/2}=\frac{\ln2}{k}$$

The maximum concentration (C_max_) and time to maximum concentration (T_max_) were obtained by visual observation of plasma or CSF concentration-time profiles. The theoretical concentration at time zero was extrapolated from initial measured concentrations. The area under the concentration-time curve to the last measured concentration (AUC_last_) was calculated by the trapezoidal method. Absolute bioavailability was calculated as AUC_last_ of PO administration group divided by AUC_last_ of IV administration group as following Equation (2), [13]:

(2) $$\mathrm{Absolute}\;\mathrm{bioavailability}\;\left(\%\right)=\frac{{\mathrm{AUC}}_{\mathrm{PO}}}{{\mathrm{AUC}}_{\mathrm{IV}}}\times \frac{{\mathrm{Dose}}_{\mathrm{IV}}}{{\mathrm{Dose}}_{\mathrm{PO}}}\times 100$$

All PK parameters were calculated as mean ± standard deviation (SD).

### 2.8. Statistical Analysis

Statistical significance was tested using Wilcoxon rank summation test with R software (R Foundation for Statistical Computing, Vienna, Austria) with *p* < 0.05 inferring significant difference.

## 3. Results and Discussion

### 3.1. Evaluation of Antioxidant Activity for TEJ-1704

**Figure 2:**
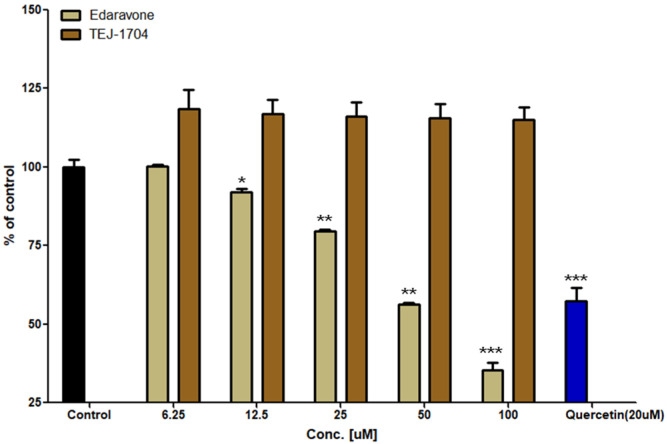
Evaluation of in vitro antioxidant activity for edaravone and TEJ-1704 (*: *p* < 0.05; **: *p* < 0.01; ***: *p* < 0.001).

As a results of the DPPH assay, the antioxidant efficacy of quercetin (positive control) was 42.6%. Edaravone showed in vitro antioxidant effects proportional to the concentration in the range of 6.25 to 100 μM. TEJ-1704 did not show in vitro antioxidant activity at any concentration. Results of DPPH assays for edaravone and TEJ-1704 are shown in Figure 2.

**Figure 3:**
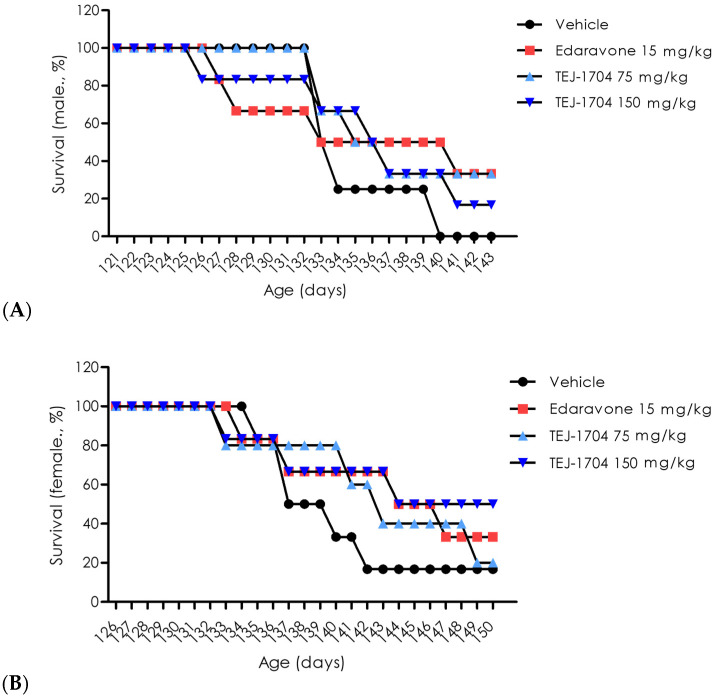
Evaluation of survival rate for edaravone and TEJ-1704. Male rats (**A**) and female rats (**B**).

The in vivo efficacy study was conducted using SOD1 mice with motor function deficits. The edaravone-administered group had an increased survival rate in both males and females compared with the vehicle-administered group. The TEJ-1704-administered group showed a similar or higher survival rate than the vehicle-administered group, and results are presented in Figure 3. In addition, the survival rate of male groups was generally lower than that of female groups because the motor function of male groups deteriorated rapidly from the onset of motor function deficit.

### 3.2. Analytical Method Development and Validation

There were no significant interferences from endogenous substances around retention time of analytes in blank matrices, indicating sufficient specificity of the method. Linearities for edaravone in rat plasma and six different tissues were excellent over concentration ranges of 2 to 2000 ng/mL. Linearities for edaravone in beagle dog plasma, cerebrospinal fluid, urine, and feces were also confirmed over the concentration ranges of 2 to 2000 ng/mL. Calibration curves fitted well, with all correlation coefficient (r^2^) exceeding 0.99. Equations for calibration curves are presented in Table 3. LLOQ of edaravone was 2 ng/mL in all matrices, which was sufficient for further pharmacokinetic studies of edaravone in rats and beagle dogs.

Intra- and inter-batch precision and accuracies for edaravone at LLOQ (2 ng/mL) and QC samples at 6, 800, and 1600 ng/mL in rat and beagle dog matrices are summarized in Table 4. As presented in the table, intra- and inter-batch precision (CV, %) and accuracy (%) data of edaravone for LLOQ and QC samples were all within ±15% for QC samples (±20% for LLOQ). Thus, the developed method satisfied the acceptance criteria.

### 3.3. Pharmacokinetic Study

**Figure 4:**
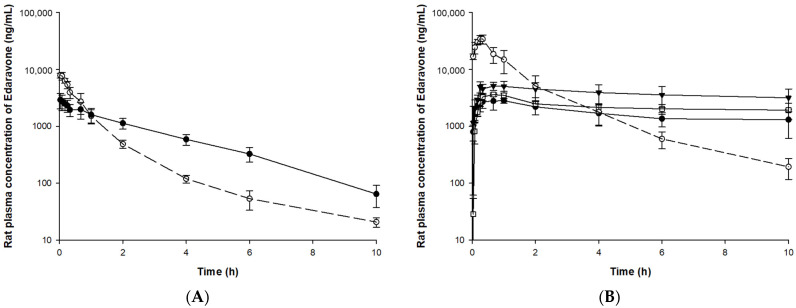
Plasma concentration of edaravone after administration of edaravone or TEJ-1704 to rats. (**A**) IV administration of edaravone 3 mg/kg (○, dotted line) and TEJ-1704 19.8 mg/kg (●, solid line). (**B**) PO administration of edaravone 30 mg/kg (○, dotted line) and TEJ-1704 99 mg/kg (●, solid line), 198 mg/kg (□, solid line), and 660 mg/kg (▼, solid line).

**Figure 5:**
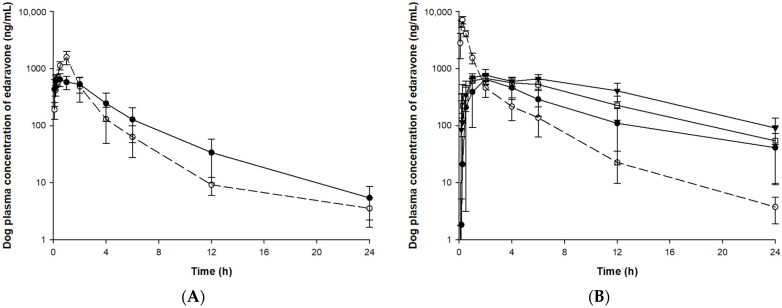
Plasma concentration of edaravone after administration of edaravone or TEJ-1704 to beagle dogs. (**A**) IV infusion of edaravone 1 mg/kg (○, dotted line) and IV bolus of TEJ-1704 3.3 mg/kg (●, solid line). (**B**) PO administration of edaravone 3 mg/kg (○, dotted line) and TEJ-1704 9.9 mg/kg (●, solid line), 19.8 mg/kg (□, solid line), and 39.6 mg/kg (▼, solid line).

The validated bioanalysis method was applied to pharmacokinetic, tissue distribution, and excretion studies after IV or PO administration of edaravone or TEJ-1704 to animals. The mean (±SD) plasma concentration-time curves of edaravone in rat and beagle dog plasma samples are illustrated in Figure 4 and Figure 5, respectively.

Since TEJ-1704 is a prodrug, it takes time to be metabolized to edaravone even after IV injection. Comparing IV administration groups of TEJ-1704 in rats and beagle dogs (group R3 and B3), the absorption phase was not observed in rats but observed in beagle dogs. These results showed evidence that TEJ-1704 was already metabolized and converted to edaravone in rats before the initial blood sampling time (about 2–5 min). TEJ-1704 is probably metabolized by carboxylesterase (CES), and since rats have CES in plasma but dogs do not have CES in plasma, it is thought that there is a difference in the absorption phase between rats and dogs [14]. Moreover, from visual observation, the IV administration of TEJ-1704 had a lower C_max_ than the IV administration of edaravone and the exposure was similar. The oral administration groups of TEJ-1704 in rats and beagle dogs also had a lower C_max_ than oral administration of edaravone at all doses. Pharmacokinetic parameters including half-life, C_0_, C_max_, T_max_, and AUC_last_ for each group are summarized in Table 5.

For SD rats, 3 mg/kg edaravone (group R1) and 19.8 mg/kg TEJ-1704 (group R3) were administered intravenously. The half-life and AUC_last_ of edaravone in both groups were not significantly different. In linear pharmacokinetics, the elimination half-life is the same regardless of the administration routes and defined by the intrinsic clearance of the drug [16]. The elimination half-life of edaravone IV administration group (group R1) and PO administration group (group R2) were estimated similarly. In the case of IV administration group of TEJ-1704 (group R3), TEJ-1704 was rapidly metabolized and converted to edaravone. Since the half-life of group R3 was not significantly different from that of the IV administration group of edaravone (group R1), it could be considered that all TEJ-1704 was converted to edaravone and only elimination occurred same as group R1. However, the elimination half-life for administration groups of TEJ-1704 (group R4–R6) was 12.9–16.5 h (no significant difference between the three groups), which was estimated to be about six times longer than that of group R1. It is thought that the absorption of edaravone (or the in vivo conversion from TEJ-1704 to edaravone) was not yet complete and may affect the elimination. Moreover, based on the similar half-lives of group R4–R6, it is considered that the saturation (or nonlinear pharmacokinetics) did not occur during the elimination process of edaravone.

In group R4–R6, the T_max_ was not significantly different from the oral administration group of edaravone (group R2). The AUC_last_ of the oral administration group of edaravone (group R2) was calculated to be twice as high as that of the oral administration group of TEJ-1704 (group R4), which loaded the same dose of edaravone. Only the AUC_last_ calculated from the oral administration group of high-dose TEJ-1704 (group R6) showed the most similar value to the AUC_last_ of the oral administration group of edaravone (group R2). The C_max_ and AUC_last_ showed no statistically significant differences between the low-dose PO group (group R4) and the medium-dose PO group (group R5) despite the doubling of the dose. Similarly, C_max_ and AUC_last_ differences between low (group R4) and high (group R6) dose groups were less than proportional compared to their administered doses. From these results, it could be inferred that saturation occurred in the absorption phase of TEJ-1704 or edaravone (to be precise, in vivo conversion process from TEJ-1704 to edaravone).

If an enzyme that metabolizes TEJ-1704 to edaravone is saturated, it will continue to produce edaravone until all TEJ-1704 is depleted. Therefore, it could explain the results of the longer elimination half-life in the oral administration group of TEJ-1704 (group R4-R6). It is necessary to identify the metabolic enzyme that converts TEJ-1704 to edaravone in vivo through an additional biological conversion study.

If both edaravone and TEJ-1704 follow linear pharmacokinetics in rats, the absolute bioavailability was calculated to be 75.22% for edaravone-administered group (calculated from group R1 and R2) and 46.10% for TEJ-1704-administered group (calculated from group R3 and R4). The absolute bioavailability calculated from edaravone-administered groups concurred with the previous report [17] that manifested high absolute bioavailability (50–90%) at dose ranges of 0.5 to 27 mg/kg. However, since the absolute bioavailability of edaravone in the TEJ-1704-administered group was lower than the value reported in the literature, a dose adjustment of the prodrug is needed.

In beagle dogs, 1 mg/kg edaravone (group B1) and 3.3 mg/kg TEJ-1704 (group B3) were administered intravenously. The half-life and AUC_last_ of edaravone in both groups were not significantly different. In group B3, the TEJ-1704 was metabolized to edaravone more slowly than in rats, showing a T_max_ of 20 min. When equivalent amounts of edaravone (group B2) and TEJ-1704 (group B4) were administered orally, the AUC_last_ was about 1.5 times the difference between the two groups, although the C_max_ was about 10 times lower in group B4 than group B2. The general trend of TEJ-1704 having lower C_max_ than the control group could be due to a characteristic of the prodrug. Although TEJ-1704 had lower C_max_, its AUC_last_ was often equal to or greater than that of the control group since TEJ-1704 might be continually metabolized to edaravone in vivo. The absolute bioavailability of TEJ-1704-administered group was calculated to be 51.82% similar to that in rats. The medium-dose PO group (group B5) had a similar AUC_last_ to the oral administration group of edaravone, but the C_max_ was calculated to be about 11 times lower.

Considering the results of rats and dogs, IV injection of TEJ-1704 instead of edaravone could improve safety because AUC_last_ was similar but C_max_ was reduced. However, in the case of the PO group, the absolute bioavailability calculated from the edaravone-administered group and TEJ-1704-administered group differs by about two times even if the equivalent dose of edaravone is the same. Therefore, based on the edaravone equivalent dose, the administration dose of TEJ-1704 should be increased by about two times or more to achieve the same in vivo exposure as the control group. In future studies, dose adjustment of TEJ-1704 is required considering absolute bioavailability in rats or dogs.

### 3.4. Distribution and Excretion

**Figure 6:**
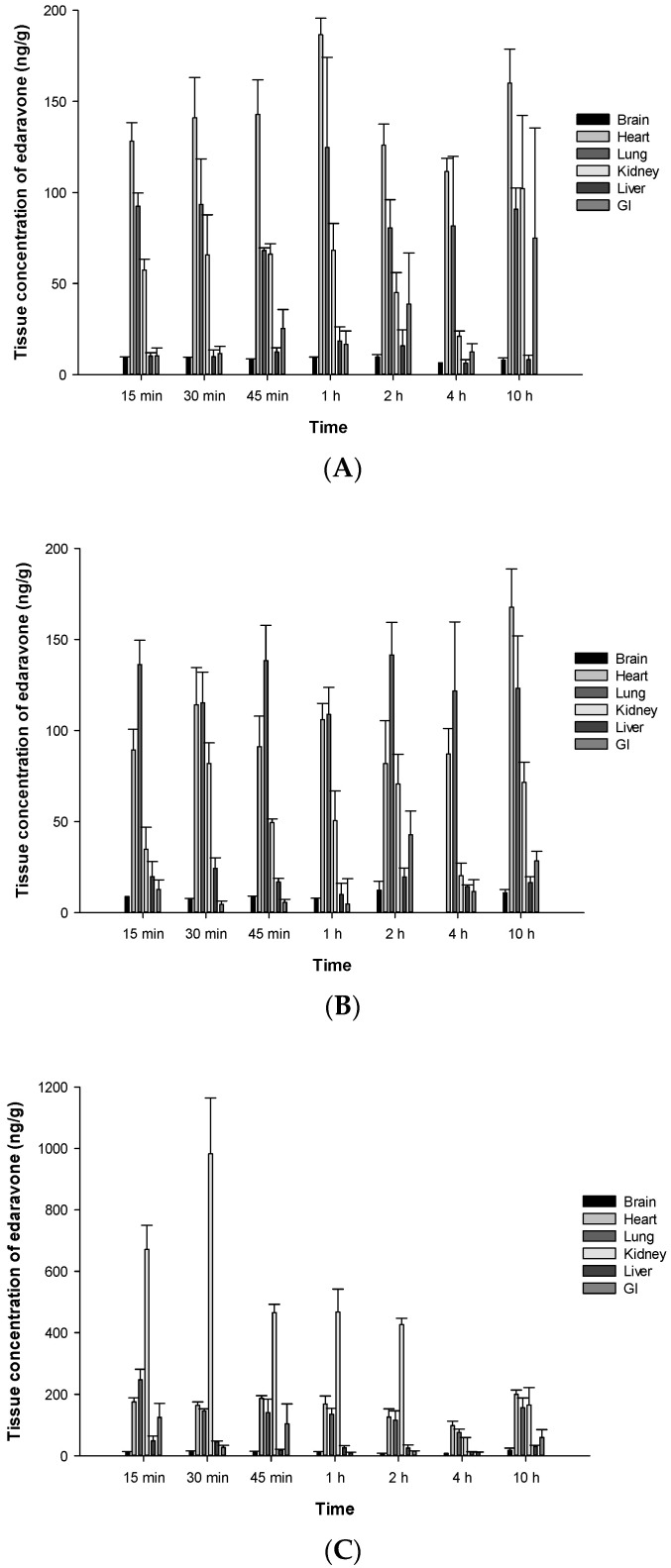
Mean concentrations (ng/g) of edaravone in various organs (brain, heart, lung, kidney, liver, GI) at different time points after oral administration of TEJ-1704 at 9.9 mg/kg (**A**), 198 mg/kg (**B**), and 660 mg/kg (**C**) to SD rats.

The organ distribution of edaravone in male SD rats was investigated following a single oral dose of TEJ-1704. Temporal mean concentrations (ng/g) of edaravone in six organs including brain, heart, lung, kidney, liver, and GI tract after the administration of TEJ-1704 at low, medium, and high doses are presented in Figure 6.

According to FDA pharmacological review of edaravone [18], ^14^C-edaravone was partitioned to aorta and kidney after it was administered intravenously to male and female Wistar rats. Brain concentrations of edaravone were 6% that of plasma at five minutes after dosing. After IV bolus administration of edaravone, it was observed that the drug was predominantly excreted into urine in both rats (61.04%) and beagle dogs (76.44%).

Edaravone showed extensive partition to the heart, lung, and kidney after oral administration of TEJ-1704 at low, medium, and high doses. Further study is required to understand the partition of edaravone in brain after oral administration of TEJ-1704. 

**Figure 7:**
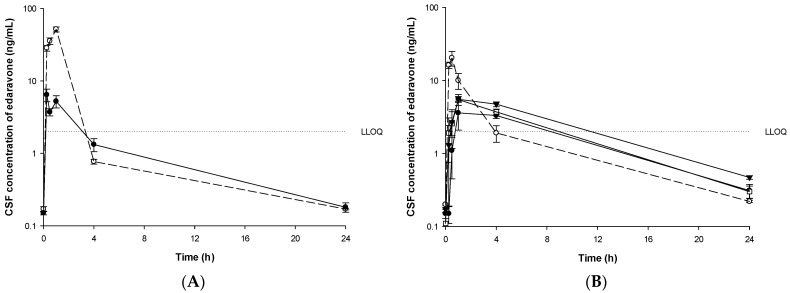
Mean CSF concentrations of edaravone after administration of edaravone or TEJ-1704 to beagle dogs. (**A**) IV infusion of edaravone 1 mg/kg (○, dotted line) and IV bolus of TEJ-1704 3.3 mg/kg (●, solid line). (**B**) PO administration of edaravone 3 mg/kg (○, dotted line) and TEJ-1704 9.9 mg/kg (●, solid line), 19.8 mg/kg (□, solid line), and 39.6 mg/kg (▼, solid line).

In beagle dogs, concentrations of edaravone in CSF, urine, and feces were investigated. Mean CSF concentrations of edaravone in groups B1-B6 are depicted in Figure 7. CSF PK parameters are summarized in Table 6.

In the IV infusion group (group B1 and B3), the CSF concentration of edaravone all disappeared within four hours after dosing and was detected below the LLOQ (2 ng/mL). In the PO administered group of edaravone (group B2), the CSF concentration disappeared in about four hours. In the PO administered group of TEJ-1704 (group B4–B6), the CSF concentration of edaravone disappeared within 8–24 h.

When 3 mg/kg edaravone was administered orally (group B2), the C_max_ and AUC_0–24h_ halved compared to those of edaravone 1 mg/kg IV infusion group (group B1). As for TEJ-1704 oral administration groups (B4–B6), the C_max_ and AUC_0–24h_ did not show a dose-proportional increase. The maximum percentages of edaravone CSF to plasma for groups B1-B6 are 4.52, 0.65, 1.00, 0.92, 0.90, and 1.14%, respectively.

Comparable literature data showed that the half-life of elimination and the AUC after intravenous injection of 3.75 mg/kg edaravone in rats were 0.61 h and 8.76 μg/mL·h [5]. The recovery cumulative excretion of edaravone after intravenous administration of 1 mg/kg in urine, bile, and feces were 0.30, 0.48, and 0.017%, respectively. According to Li et al. [7], the peak concentration of edaravone in rat organ tissues (kidney, heart, brain, spleen, and liver) occurred at 30 min after the intravenous administration of 1 mg/kg edaravone and the highest concentrations of edaravone were detected in the kidney. In beagles, Shao et al. [10] reported edaravone half-life of about 9 h and AUC_0–24h_ of about 26,694 ng/mL·h, 42,090 ng/mL·h, and 55,811 ng/mL·h after intravenous administration of 2.4, 4.8, and 9.6 mg/kg edaravone, respectively. According to EMA assessment report [19], 75% to 85% of the administered ^14^C-edaravone was excreted via the renal route within 24 h while only a minor fraction was found in feces in all animal species including rats, monkeys, and dogs. The report concurred with the FDA pharmacological review which reported that 61–83% of the administered ^14^C-edaravone was excreted via urine in rat, dog, and monkeys [18]. However, from the excretion studies of the parent drug, edaravone was almost equally excreted via urine and feces after oral administration of TEJ-1704. Specifically, edaravone (parent drug) was excreted mostly via urine until 12 h and then via feces from 12 h after the administration of TEJ-1704 (prodrug). Based on results after the administration of TEJ-1704, about 3% of edaravone was excreted through urine and feces. This suggests the possibility that all TEJ-1704 were not converted to edaravone and were excreted as unchanged prodrugs due to either the GI absorption of TEJ-1704 was saturated or the saturation of the enzyme that metabolizes TEJ-1704 to edaravone. Therefore, further studies such as the absorption process of TEJ-1704 and pharmacokinetics of the unchanged prodrug (TEJ-1704) are needed for the calculation of metabolic conversion ratio and the dose adjustment of TEJ-1704.

## 4. Conclusions

A modified analysis method developed for the quantitation of edaravone in rat and beagle dog matrices after PO or IV administration of edaravone or TEJ-1704 was validated and successfully applied to pharmacokinetic, distribution, and excretion studies of TEJ-1704. Plasma pharmacokinetics and CSF pharmacokinetics were estimated using non-compartmental analysis. In plasma, administration of TEJ-1704 resulted in greater AUC_last_ than administration of edaravone. However, the TEJ-1704-administered group showed lower C_max_ and AUC_0–24h_ than the edaravone-administered group. After oral administration of TEJ-1704 to rats, edaravone was found mainly in the heart, lung, and kidney. After oral administration of TEJ-1704 to beagle dogs, edaravone was excreted via urine and feces in approximately equal amounts. Further studies on the characteristics of TEJ-1704 are needed to fully understand and interpret the underlying causes for the difference between the novel prodrug and edaravone. The metabolic conversion rate or excretion rate of TEJ-1704 can be confirmed through metabolic enzyme studies and pharmacokinetic studies of TEJ-1704 itself. Based on these data, it is expected that a metabolic PK model can be built and used for the dose adjustment of the prodrug.

## Figures and Tables

**Table 1:** The pharmacokinetic study design using Sprague Dawley rats.

Group	Drug	Route	*n*	Dose (mg/kg)	Edaravone Equivalent Dose (mg/kg)	Blood Sampling Time	Organ Collection
R1	Edaravone	IV	5	3	3	0, 2, 5, 10, 15, 25, 40 min 1, 2, 4, 6, 10 h	-
R2	Edaravone	PO	5	30	30		-
R3	TEJ-1704	IV	5	19.8	6		-
R4	TEJ-1704	PO	5	99	30		10 h
R5	TEJ-1704	PO	5	198	60		10 h
R6	TEJ-1704	PO	5	660	200		10 h
R7	TEJ-1704	PO	18	99	30	-	15, 30, 45 min 1, 2, 4 h
R8	TEJ-1704	PO	18	198	60	-	
R9	TEJ-1704	PO	18	660	200	-	

**Table 2:** The pharmacokinetic study design using beagle dogs (*n* = 5).

Group	Drug	Route	Dose (mg/kg)	Edaravone Equivalent Dose (mg/kg)	Sampling Time
B1	Edaravone	IV infusion (1 h)	1	1	Blood: 0, 2, 5, 10, 15, 25, 40 min, 1, 2, 4, 6, 10 h CSF: 15, 30 min, 1, 4, 24 h Urine/Feces (B3–B6): 0–2, 2–6, 6–12, 12–24 h
B2	Edaravone	PO	3	3	
B3	TEJ-1704	IV bolus	3.3	1	
B4	TEJ-1704	PO	9.9	3	
B5	TEJ-1704	PO	19.8	6	
B6	TEJ-1704	PO	39.6	12	

**Table 3:** Calibration information of edaravone in rat and beagle dog matrices (*n* = 5).

Matrix	Linear Regression Equation *	r^2^
Rats		
Plasma	y = (0.016 ± 0.001) + (0.002 ± 0.003)	0.998 ± 0.001
Brain	y = (0.020 ± 0.02) + (0.006 ± 0.003)	0.996 ± 0.003
Liver	y = (0.020 ± 0.001) + (0.046 ± 0.008)	0.999 ± 0.001
Kidney	y = (0.019 ± 0.001) + (0.043 ± 0.024)	0.998 ± 0.001
Heart	y = (0.019 ± 0.001) + (0.013 ± 0.003)	0.996 ± 0.005
Lung	y = (0.020 ± 0.01) + (0.056 ± 0.019)	0.997 ± 0.002
Gastro-intestinal (GI) tract	y = (0.020 ± 0.01) + (0.026 ± 0.005)	0.997 ± 0.002
Beagle dogs		
Plasma	y= (0.023 ± 0.001) + (0.009 ± 0.008)	0.998 ± 0.002
Cerebrospinal fluid (CSF)	y= (0.023 ± 0.001) + (0.002 ± 0.001)	0.999 ± 0.001
Urine	y= (0.016 ± 0.001) − (0.063 ± 0.021)	0.991 ± 0.0004
Feces	y= (0.022 ± 0.001) − (0.155 ± 0.252)	0.995 ± 0.001

* In linear regression equations, y is the peak-area ratio of analytes to IS and x (ng/mL) is the plasma concentration of analyte.

**Table 4:** Precision and accuracy for edaravone bioanalysis method in rat and beagle dog matrices (*n* = 5).

Matrix	Intra-Batch								Inter-Batch							
	Precision (CV, %)				Accuracy (%)				Precision (CV, %)				Accuracy (%)			
	LLOQ	QL	QM	QH	LLOQ	QL	QM	QH	LLOQ	QL	QM	QH	LLOQ	QL	QM	QH
Rats																
Plasma	8.15	6.29	2.01	3.41	106.54	107.30	108.55	109.23	5.21	5.98	1.14	1.98	106.81	104.42	108.14	107.13
Brain	12.46	5.39	1.53	6.37	100.81	94.11	102.17	99.32	6.39	2.62	1.65	2.83	108.61	96.59	102.5	102.66
Liver	7.32	6.35	1.04	0.33	110.88	98.25	97.65	94.83	7.75	2.85	4.02	2.83	106.59	100.82	101.57	97.91
Kidney	9.19	1.71	1.48	7.37	107.35	102.92	98.11	98.00	2.00	7.20	2.85	2.07	104.95	99.30	100.85	99.30
Heart	7.25	7.02	2.07	2.88	111.55	96.61	101.66	101.15	1.70	10.28	3.87	3.28	111.22	100.28	101.27	102.98
Lung	5.39	7.92	2.37	2.4	99.45	96.87	99.06	95.25	9.96	3.48	5.74	7.41	100.12	95.59	100.56	100.8
GI tract	2.72	6.26	3.12	2.67	109.85	99.38	97.69	104.59	12.08	11.28	0.23	2.01	107.25	101.87	100.97	105.42
Beagle dogs																
Plasma	5.57	2.27	2.87	1.36	106.42	103.73	98.14	101.36	2.84	3.95	3.42	2.00	104.28	101.26	99.19	102.67
CSF	8.58	4.18	2.78	3.08	107.81	102.99	98.54	100.34	3.12	2.51	0.25	2.61	100.83	105.10	98.97	103.17
Urine	10.57	5.56	6.38	5.83	88.88	92.89	103.83	104.76	15.24	10.17	0.23	3.53	99.53	87.17	102.45	109.44
Feces	0.35	2.01	5.45	0.22	110.72	101.32	105.36	98.30	7.16	11.34	9.35	9.06	111.17	98.97	98.73	98.55

**Table 5:** Summary of the edaravone plasma PK parameters in rats and beagle dogs.

Group	Route	Half-Life [15]	T_max_ [15]	C_0_ (ng/mL)	C_max_ (ng/mL)	AUC_last_ (ng·h/mL)
Rats						
R1	IV	1.8 ± 0.1	-	8398.7 ± 1543.1	-	6204.1 ± 1026.0
R2	PO	1.7 ± 0.2	0.4 ± 0.1	-	35,420.4 ± 4878.3	46,667.0 ± 12,543.6
R3	IV	1.8 ± 0.2	0.04 ± 0.02		2960.4 ± 808.2	6948.4 ± 1499.9
R4	PO	12.9 ± 4.8	0.7 ± 0.3	-	3285.6 ± 456.9	16,016.7 ± 3436.1
R5	PO	15.0 ± 8.4	0.8 ± 0.2	-	3866.6 ± 427.1	20,497.9 ± 6365.7
R6	PO	16.5 ± 11.5	0.9 ± 0.7	-	5680.5 ± 850.7	38,596.6 ± 11,716.4
Beagle dogs						
B1	IV	4.1 ± 0.5	-	-	1589.6 ± 415.0	3120.1 ± 1041.1
B2	PO	3.6 ± 0.4	0.2 ± 0.1	-	7840.7 ± 1144.4	7284.6 ± 1641.1
B3	IV	4.0 ± 0.3	0.3 ± 0.2	-	663.6 ± 154.4	3014.7 ± 1142.6
B4	PO	5.9 ± 1.1	1.8 ± 0.4	-	665.2 ± 125.0	4686.4 ± 2092.4
B5	PO	5.4 ± 1.6	1.6 ± 0.5	-	712.9 ± 101.2	7258.9 ± 1727.6
B6	PO	6.2 ± 1.4	1.3 ± 0.7	-	809.2 ± 169.3	9953.7 ± 2135.0

**Table 6:** Summary of edaravone CSF PK parameters in beagle dogs.

Group	T_max_ [15]	C_max_ (ng/mL)	AUC_0–24h_ (ng·h/mL)
B1	1.0 ± 0.0	51.7 ± 9.2	121.4 ± 19.5
B2	0.4 ± 0.1	23.5 ± 7.1	53.4 ± 9.9
B3	0.3 ± 0.0	6.4 ± 2.8	29.1 ± 11.0
B4	2.2 ± 1.6	4.9 ± 2.0	48.0 ± 8.0
B5	0.8 ± 0.3	6.4 ± 1.5	56.8 ± 11.0
B6	1.6 ± 1.6	5.6 ± 1.1	66.9 ± 9.7

## Data Availability

Not applicable.
